# Supplementary material for: Molecular mechanisms of ion conduction and ion selectivity in TMEM16 lipid scramblases
Source: Nat Commun. 2021 May 14;12:2826. doi: 10.1038/s41467-021-22724-w (PMC8121942; doi:10.1038/s41467-021-22724-w)
Supplement: Supplementary file 1 — Supplementary Information [file 41467_2021_22724_MOESM1_ESM.pdf]

Supplementary Information

Molecular mechanisms of ion conduction and ion selectivity in TMEM16 lipid scramblases

Andrei Y. Kostritskii and Jan-Philipp Machtens

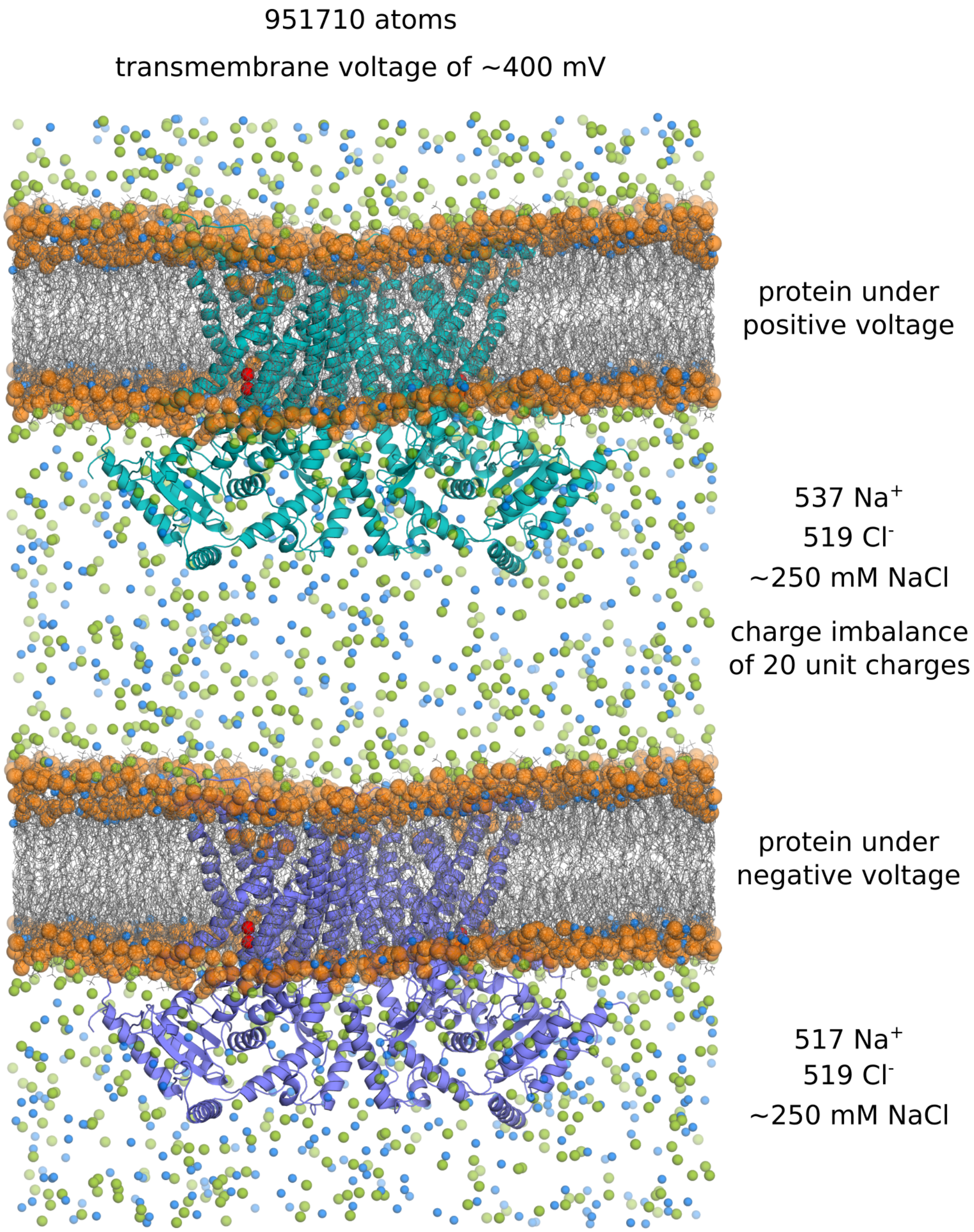

Supplementary Fig. 1: **Computational electrophysiology setup.** Setup of a double bilayer simulation used to study ion conduction by the CompEL method. Two copies of nhTMEM16 were embedded into the lipid bilayers in a parallel orientation, with the top protein (teal) subjected to positive voltages and the bottom protein (violet) to negative voltages via the higher number of Na^+ ions in the middle compartment. In such system, a charge imbalance of 20 elementary charges gave rise to a transmembrane voltage of about 400 mV. Phosphorus atoms and POPC lipid tails are shown as orange spheres and gray lines, respectively. Na^+, Cl^-, and Ca^{2+} ions are shown as green, blue, and red spheres, respectively. Water is omitted for clarity.

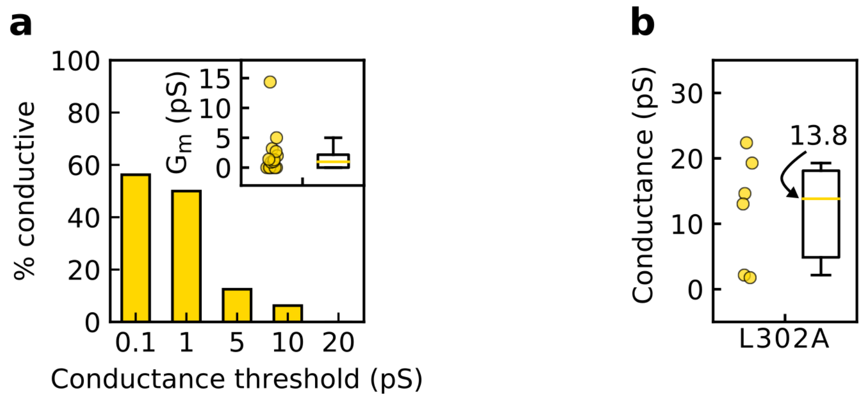

Supplementary Fig. 2: **Ion conduction of the L302A mutant of nhTMEM16 in a POPC membrane.** **a** Dependence of the percentage of conductive protomers in simulations on the conductance threshold used to distinguish between conductive and nonconductive states. The inset shows the mean conductance calculated globally as ratio between number of permeation events and the simulation time since a first permeation event. Data correspond to n=16 independent protomers. **b** Instantaneous conductance of the L302A mutant. Data correspond to n=6 independent protomers, median value is indicated. **a,b** Each data point represents an independent protomer, and boxplots are defined as follows: the middle line is the median, the lower and upper hinges correspond to the first and third quartiles, whiskers show the 5th and 95th percentiles.

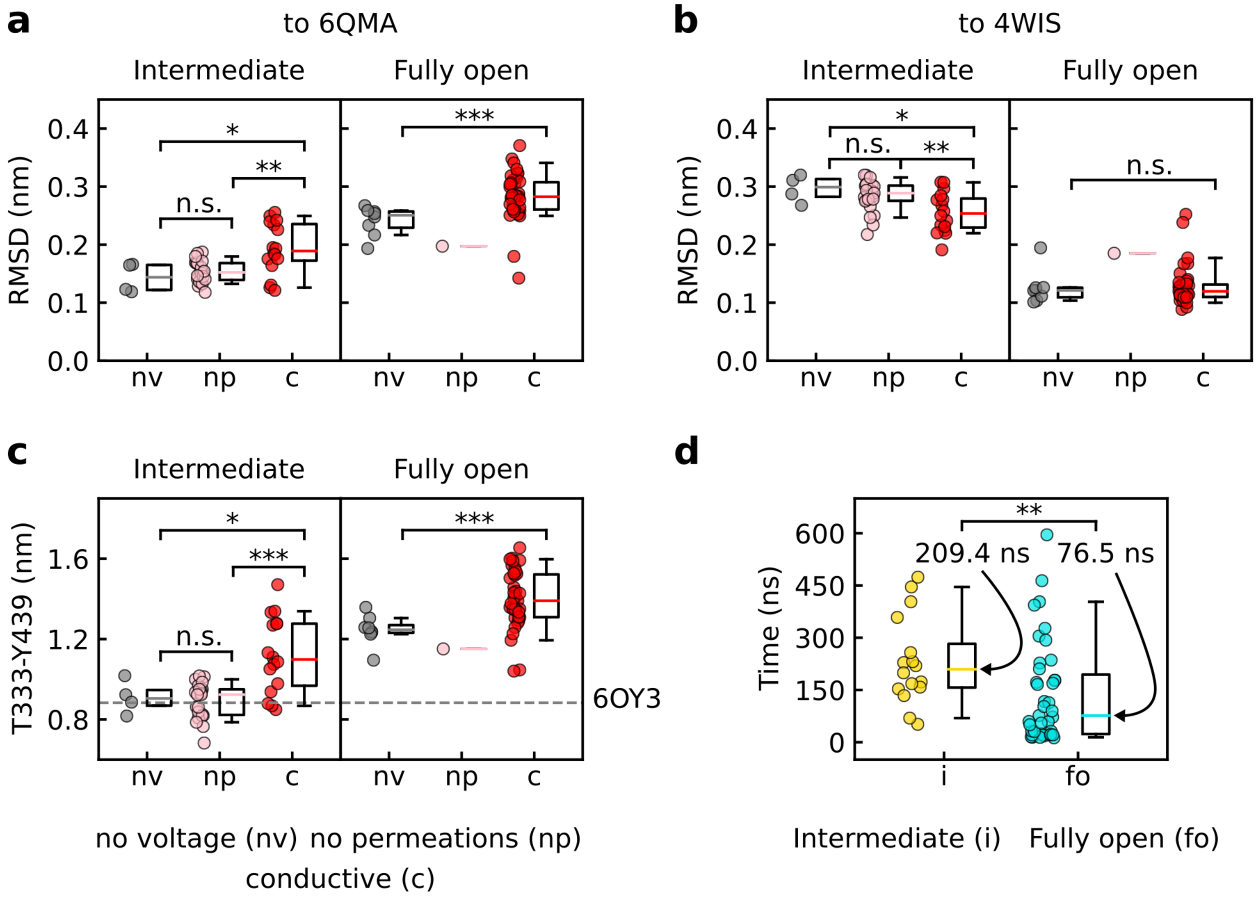

Supplementary Fig. 3: **nhTMEM16 in the intermediate conformation requires structural changes to initiate ion conduction. a,b** RMSD of Cα atoms of the residues lining the subunit cavity in protomers at zero voltage (no voltage), and in conductive and nonconductive (no permeations) protomers in simulations of the nhTMEM16 intermediate and the fully open states, calculated with respect to the 6QMA (**a**) and 4WIS (**b**) structures. **a** Intermediate: n_{nv}=4, n_{np}=23, n_c=16 independent protomers, $p_{nv,np}$=0.2, $p_{nv,c}$=0.02, $p_{np,c}$=0.002; Fully open: n_{nv}=8, n_{np}=1, n_c=39 independent protomers, $p_{nv,c}$=0.0006. **b** Intermediate: n_{nv}=4, n_{np}=23, n_c=16 independent protomers, $p_{nv,np}$=0.2, $p_{nv,c}$=0.02, $p_{np,c}$=0.009; Fully open: n_{nv}=8, n_{np}=1, n_c=39 independent protomers, $p_{nv,c}$=0.67. **c** Width of the cavity (measured as the distance between Cα atoms of T333 and Y439 residues) in the protomers in the intermediate and fully open conformations. The cavity width in the L302A structure (PDB ID: 6OY3) is indicated by a dashed line. Intermediate: n_{nv}=4, n_{np}=23, n_c=16 independent protomers, $p_{nv,np}$=0.57, $p_{nv,c}$=0.03, $p_{np,c}$=0.00011; Fully open: n_{nv}=8, n_{np}=1, n_c=39 independent protomers, $p_{nv,c}$=0.0009. **d** Time of the first permeation event detected in the protomers in the intermediate and fully open conformations. n_i=16, n_{fo}=39 independent protomers, p=0.004. Median values are labeled. **a–d** Each data point represents an independent protomer, and boxplots are defined as follows: the middle line is the median, the lower and upper hinges correspond to the first and third quartiles, whiskers show the 5th and 95th percentiles. Significance was evaluated with the Mann-Whitney test, one-sided: $p > 0.05$ (n.s.), $p < 0.05$ (*), $p < 0.01$ (**), $p < 0.001$ (****), $p < 0.0001$ (****).

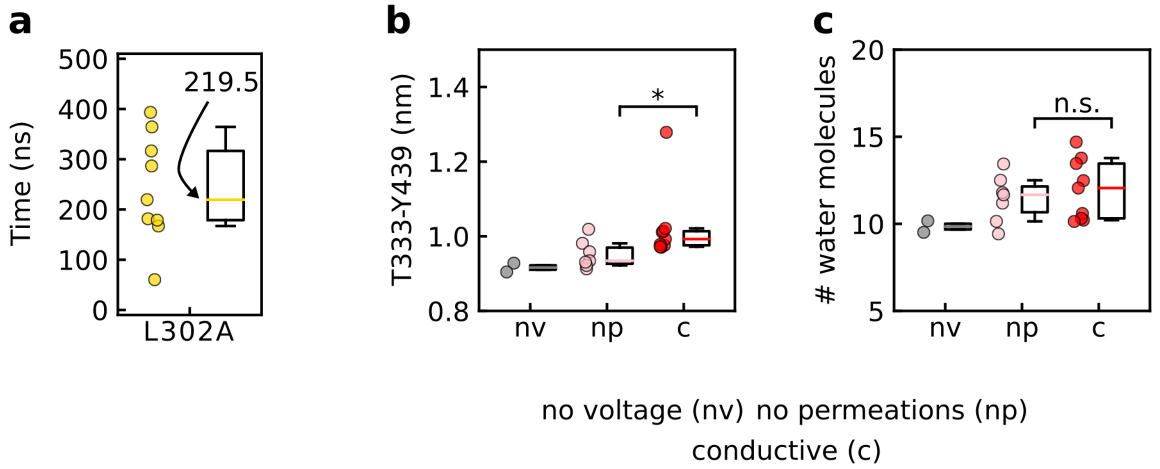

Supplementary Fig. 4: **Conductive and nonconductive states of the L302A pore. a** Time of the first permeation event detected in the L302A protomers. Data correspond to n=9 independent protomers, median value is indicated. **b** Width of the L302A cavity. The width was measured as distance between Cα atoms of T333 and Y439 residues. Data correspond to n_{nv}=2, n_{np}=7, n_c=9 independent protomers, $p_{np,c}$=0.02. **c** Hydration of the L302A pore as number of water molecules in the extracellular part of the subunit cavity. Data correspond to n_{nv}=2, n_{np}=7, n_c=9 independent protomers, $p_{np,c}$=0.3. **a–c** Each data point represents an independent protomer, and boxplots are defined as follows: the middle line is the median, the lower and upper hinges correspond to the first and third quartiles, whiskers show the 5th and 95th percentiles. Significance was evaluated with the Mann-Whitney test, one-sided: $p > 0.05$ (n.s.), $p < 0.05$ (*).

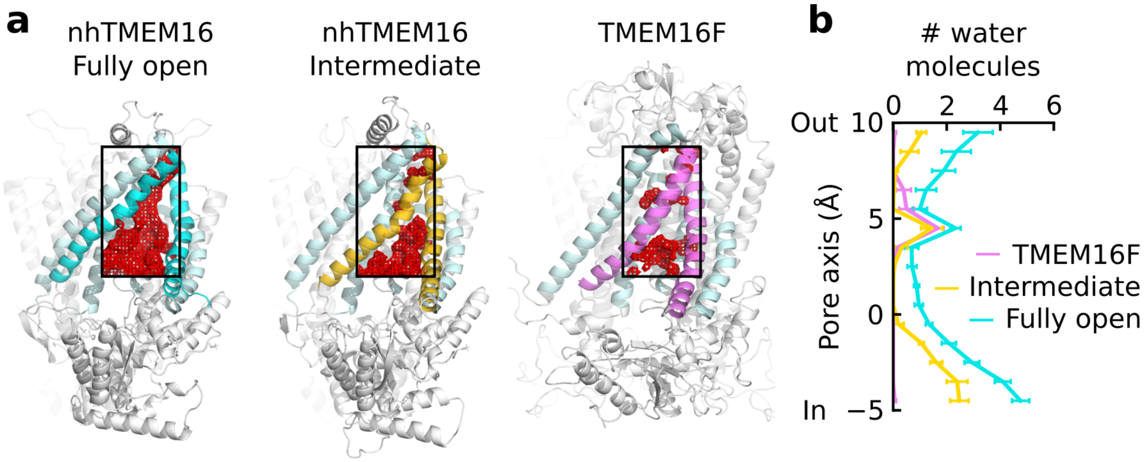

Supplementary Fig. 5: **Hydration of the nhTMEM16 and TMEM16F pores.** **a** Water density within the subunit cavities of TMEM16F and nhTMEM16 in the fully open and intermediate states is shown as meshes. Representative results from simulations with zero voltage are shown. Water density was normalized to the value in the bulk solution and contoured at 1.2σ. **b** Hydration profiles of the TMEM16F pore and of the nhTMEM16 pore in the intermediate and the fully open states, as obtained from zero-voltage simulations. Hydration is represented by the number of water molecules in 1-Å sections along the pore axis. **a,b** Hydration distributions were calculated with respect to the pore center. Average distributions across independent protomer simulations are shown, with error bars representing the standard error of mean. Data were derived from n=4, n=8, and n=6 independent protomers of nhTMEM16 in intermediate and fully open conformations, and TMEM16F, respectively.

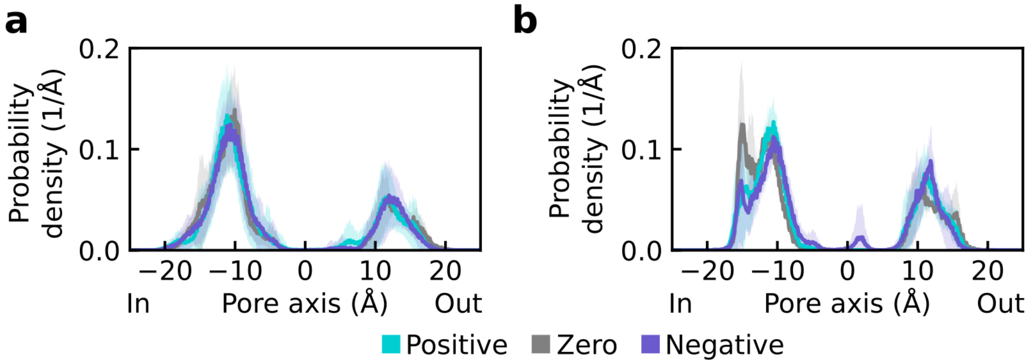

Supplementary Fig. 6: **Lipid headgroups are excluded from the pore in the intermediate state in wild-type and L302A nhTMEM16.** Local probability density distribution of POPC phosphorus atoms along the subunit cavity in the intermediate state in wild-type (**a**) and L302A nhTMEM16 (**b**). In **a** data were derived from n=4, n=13, and n=13 independent protomers at zero, positive, and negative voltages, respectively. In **b** data were derived from n=2, n=8, and n=8 independent protomers at zero, positive, and negative voltages, respectively. **a,b** Distributions were calculated with respect to the pore center. Average distributions across independent protomer simulations are shown, with shaded areas representing the standard error of mean.

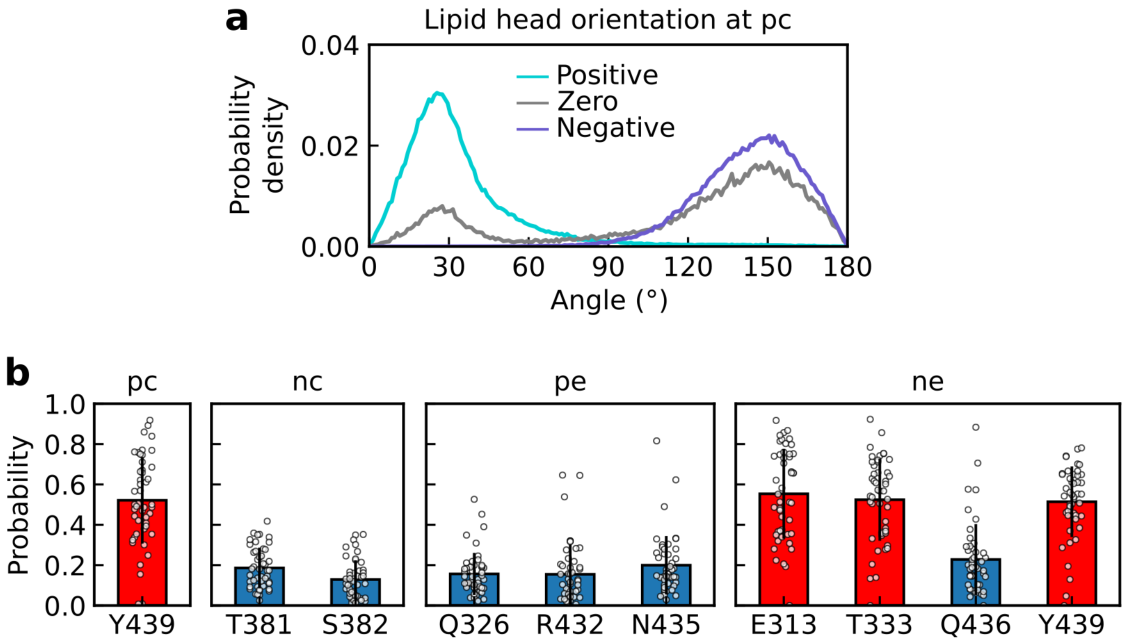

Supplementary Fig. 7: **Voltage and protein–lipid interactions define the arrangement of lipid headgroups within the nhTMEM16 subunit cavity. a** Distribution of the angle formed by a POPC headgroup located at the *pc* site with the membrane normal at positive-, negative-, and zero-voltage conditions. **b** Probability that oxygen or nitrogen atoms of hydrophilic residues are in a direct contact (distance of < 5 Å) with either the phosphorus or nitrogen atoms of POPC headgroups at their localization sites within the nhTMEM16 subunit cavity. Only residues with a contact probability of > 10 % are shown (those with a contact probability of > 35 % are shown in red and all other shown in blue). Bars show mean probability, and error bars represent the standard deviation. Data correspond to n=47, n=46, n=48, and n=47 independent protomers for *pc*, *nc*, *pe*, and *ne* sites, respectively.

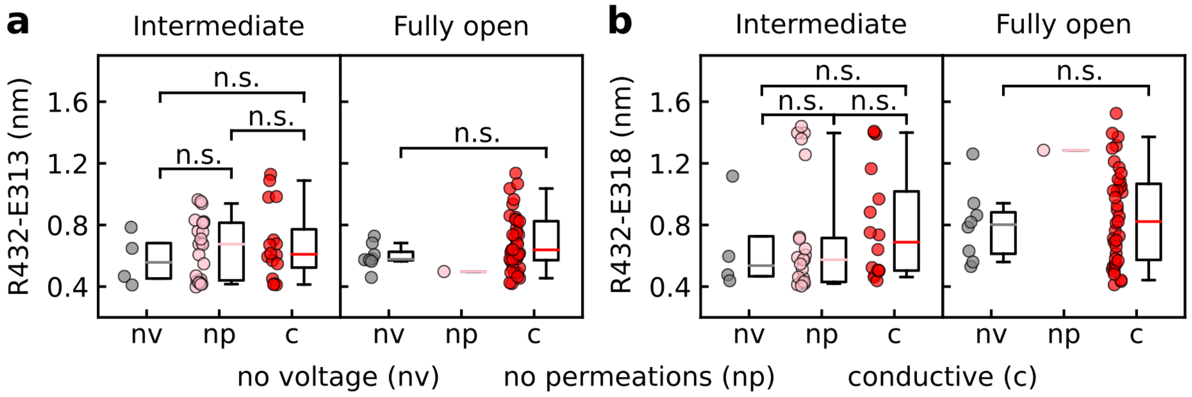

Supplementary Fig. 8: **Configuration of the E313/E318/R432 triad.** **a,b** Distance between CZ atom of R432 and CD atom of E313 (**a**) or E318 (**b**) in protomers at zero voltage (no voltage), and in conductive and nonconductive (no permeations) protomers in simulations of nhTMEM16 in the intermediate and the fully open states. **a** Intermediate: data correspond to n_{nv}=4, n_{np}=23, n_c=16 independent protomers, $p_{nv,np}$=0.4, $p_{nv,c}$=0.5, $p_{np,c}$=0.99; Fully open: data correspond to n_{nv}=8, n_{np}=1, n_c=39 independent protomers, $p_{nv,c}$=0.2. **b** Intermediate: data correspond to n_{nv}=4, n_{np}=23, n_c=16 independent protomers, $p_{nv,np}$=0.9, $p_{nv,c}$=0.4, $p_{np,c}$=0.2; Fully open: data correspond to n_{nv}=8, n_{np}=1, n_c=39 independent protomers, $p_{nv,c}$=0.7. **a,b** Each data point represents an independent protomer, and boxplots are defined as follows: the middle line is the median, the lower and upper hinges correspond to the first and third quartiles, whiskers show the 5th and 95th percentiles. Significance was evaluated with the Mann-Whitney test, two-sided: $p > 0.05$ (n.s.).

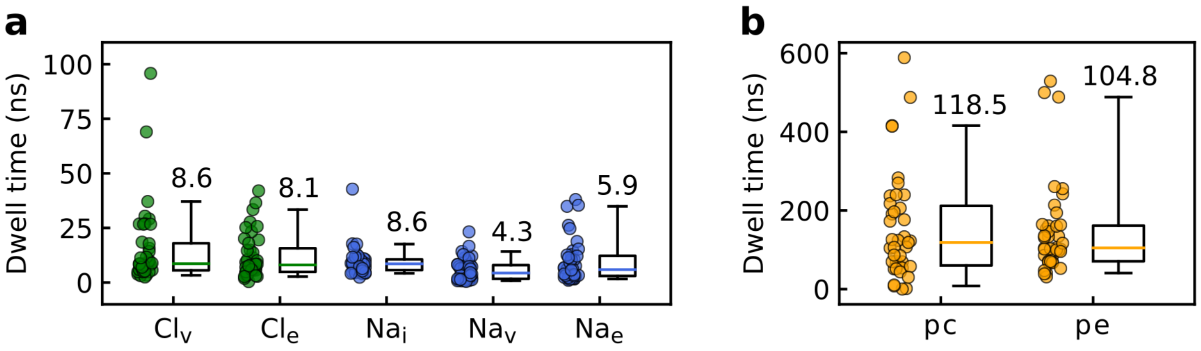

Supplementary Fig. 9: **Maximum dwell time of ions and lipid headgroups at their dwelling sites along the fully open subunit cavity of nhTMEM16 in a pure POPC membrane.** **a** Maximum dwell time of ions at the corresponding accumulation sites. Data correspond to n=40 independent protomers. **b** Maximum dwell time of the phosphates of POPC lipids at the corresponding accumulation sites. Data correspond to n=39 independent protomers. **a,b** Each data point represents an independent protomer, and boxplots are defined as follows: the middle line is the median, the lower and upper hinges correspond to the first and third quartiles, whiskers show the 5th and 95th percentiles. Median values are indicated.

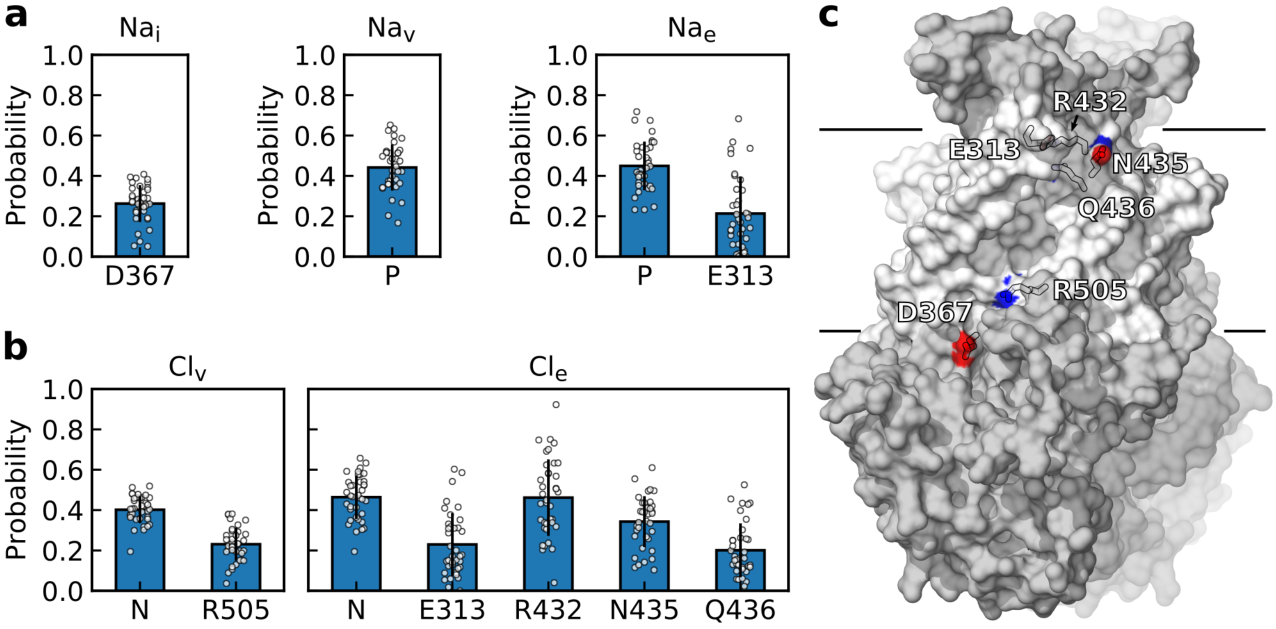

Supplementary Fig. 10: **Analysis of interaction sites of permeating ions in nhTMEM16.** **a**,**b** Probability that oxygen or nitrogen atoms of hydrophilic residues or phosphorus or nitrogen atoms of POPC headgroups are in direct contact (distance of < 5 Å) with a permeating Na^+ (**a**) or Cl^- (**b**) ion at a certain localization site (Na_i, Na_v, Na_e, Cl_v, Cl_e) within the pore. Only residues with a contact probability of > 20 % are shown. Bars show mean probability, and error bars represent the standard deviation. Data correspond to n=40 independent protomers. **c** Positions of residues indicated in the top panel are shown within the nhTMEM16 subunit cavity structure. The transmembrane part of the protein is shown in white. Black lines indicate membrane borders.

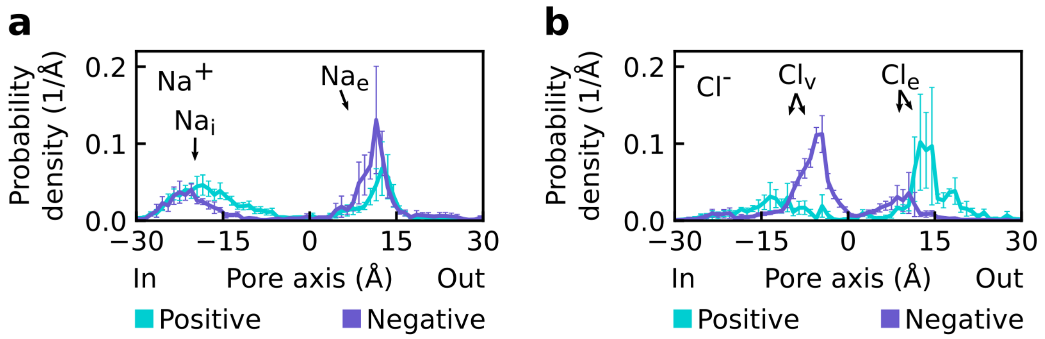

Supplementary Fig. 11: **Distribution of the permeating ions along the nhTMEM16 pore in the intermediate state.** **a,b** Probability density distributions of permeating Na^+ (**a**) and Cl^- (**b**) ions with respect to the pore center. Ion-localization sites (Na_i, Na_e, Cl_v, Cl_e) are indicated. Average distributions across independent protomer simulations are shown, with error bars representing the standard error of mean. Data were derived from n=5 and n=5 independent protomers at positive and negative voltages, respectively, in **a**; and from n=4 and n=10 independent protomers at positive and negative voltages, respectively, in **b**.

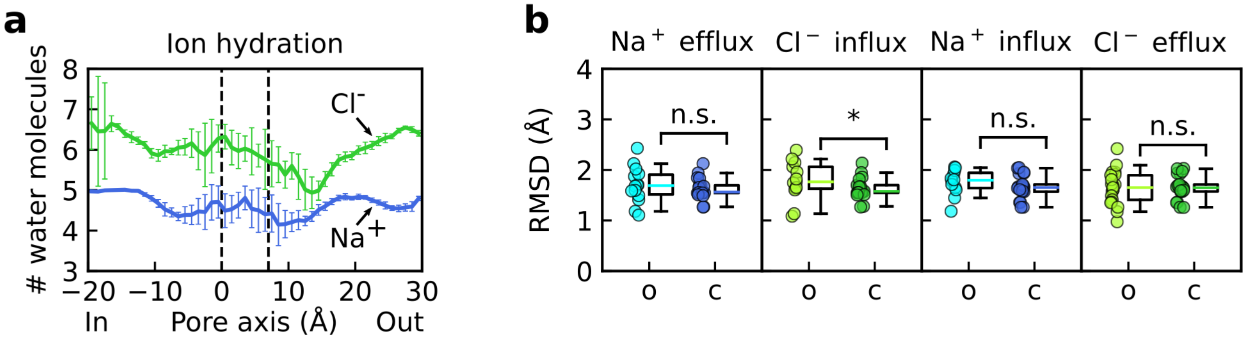

Supplementary Fig. 12: **Blockage of ions is not caused by dehydration penalty or conformational change of the nhTMEM16 subunit cavity.** **a** Distribution of number of water molecules within the first hydration shell of ions along the nhTMEM16 pore. Dashed lines indicate the neck region of the pore. Average distributions for all simulated protomers are shown, and error bars represent the standard deviation. Data were derived from n=40 independent protomers. **b** RMSD of Cα atoms of the pore-lining residues, calculated separately for the open and closed states of the pore. Each data point represents an independent protomer, and boxplots are defined as follows: the middle line is the median, the lower and upper hinges correspond to the first and third quartiles, whiskers show the 5th and 95th percentiles. Significance was evaluated with the Mann-Whitney test, one-sided: $p > 0.05$ (n.s.), $p < 0.05$ (*). The number of data points and p-values (n_o, n_c, p) for each panel (left to right) were (14, 18, 0.2), (13, 18, 0.04), (12, 21, 0.1), and (21, 21, 0.5).

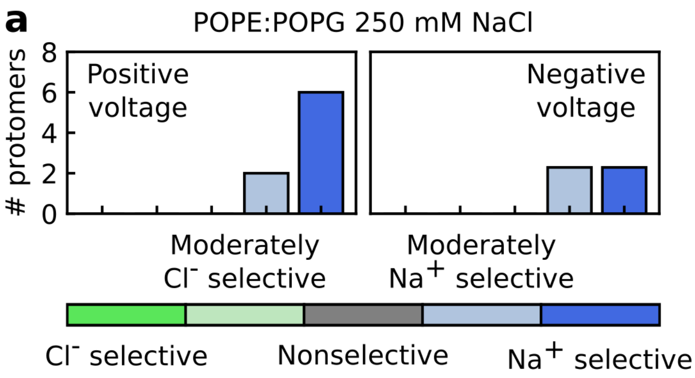

Supplementary Fig. 13: **Ion selectivity and ion conductance of nhTMEM16 in anionic membranes.** **a** Ion selectivity of nhTMEM16 protomers at positive and negative voltages in a POPE:POPG (3:1) lipid membrane. **b** Instantaneous ion conductance of nhTMEM16 in POPC:POPS and POPE:POPG membranes. Each data point represents an independent protomer, and boxplots are defined as follows: the middle line is the median, the lower and upper hinges correspond to the first and third quartiles, whiskers show the 5th and 95th percentiles. Data correspond to n=17 and n=15 independent protomers in POPC:POPS and POPE:POPG membranes, respectively.

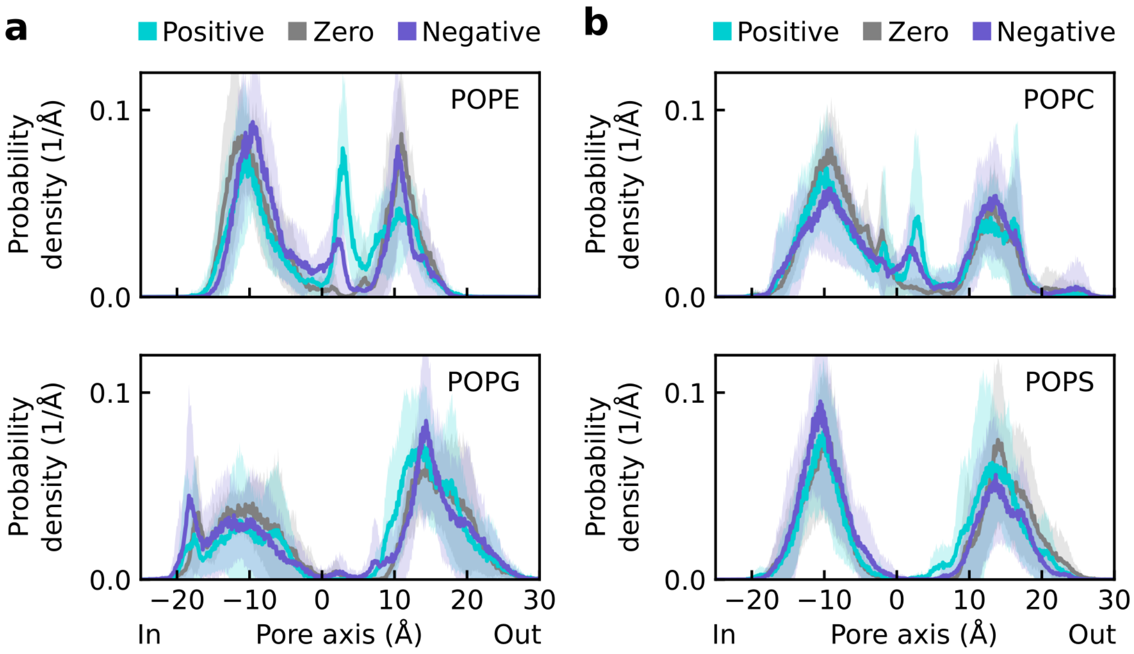

Supplementary Fig. 14: **Anionic lipids stay at the entrances to the nhTMEM16 pore.** Distribution of lipid phosphorus atoms along the nhTMEM16 subunit cavity in either a POPE:POPG (**a**) or POPC:POPS (**b**) membrane at 250 mM NaCl. Distributions were averaged over all simulated protomers and shaded areas represent the standard error of mean. Data were derived from n=6, n=12, and n=12 independent protomers at zero, positive, and negative voltages, respectively, in **a**; and from n=8, n=12, and n=12 independent protomers at zero, positive, and negative voltages, respectively, in **b**.

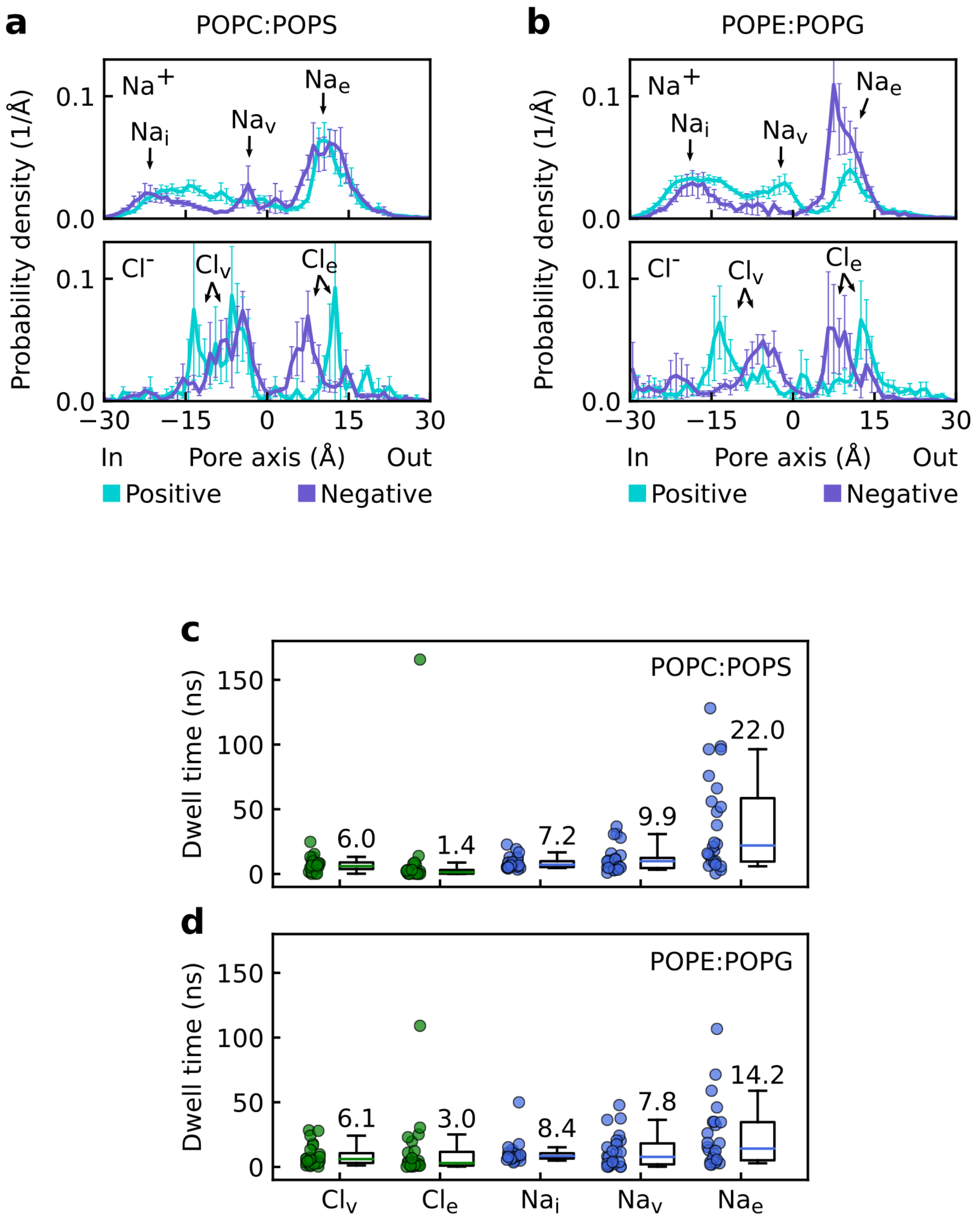

Supplementary Fig. 15: **Ion-localization sites along the proteolipidic pore in POPC:POPS and POPE:POPG membranes. a,b** Probability density distributions of permeating Na^+ (top) and Cl^- (bottom) ions along the pore in POPC:POPS (**a**) and POPE:POPG (**b**) membranes at 250 mM NaCl. Ion-localization sites (Na_i, Na_v, Na_e, Cl_v, Cl_e) are indicated. Average distributions across independent protomer simulations are shown, with error bars representing the standard error of mean. In **a**, data were derived from n=8 and n=10 independent protomers at positive and negative voltages, respectively, for Na^+; and from n=3 and n=4 independent protomers at positive and negative voltages, respectively, for Cl^-. In **b**, data were derived from n=9 and n=5 independent protomers at positive and negative voltages, respectively, for Na^+; and from n=4 and n=6 independent protomers at positive and negative voltages, respectively, for Cl^-. **c,d** Maximum dwell time of ions at the localization sites in POPC:POPS (**c**) and POPE:POPG (**d**) membranes at 250 mM NaCl. Data correspond to n=24 independent protomers in each membrane.

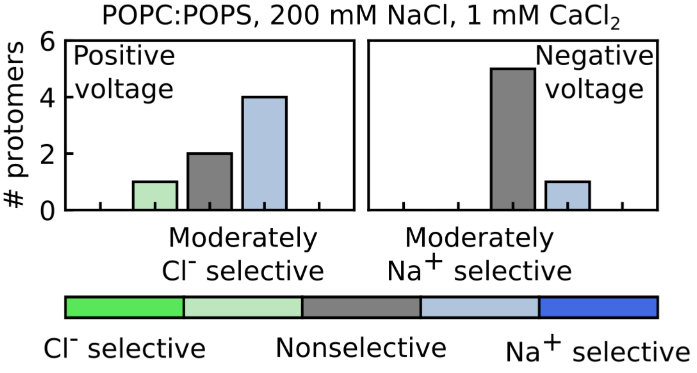

Supplementary Fig. 16: **Ion selectivity of the nhTMEM16 proteolipidic pore in POPC:POPS membrane at 1 mM $CaCl_2$.** Ion selectivity of nhTMEM16 protomers at positive and negative voltages in a POPC:POPS (1:1) lipid membrane and 1 mM $CaCl_2$.

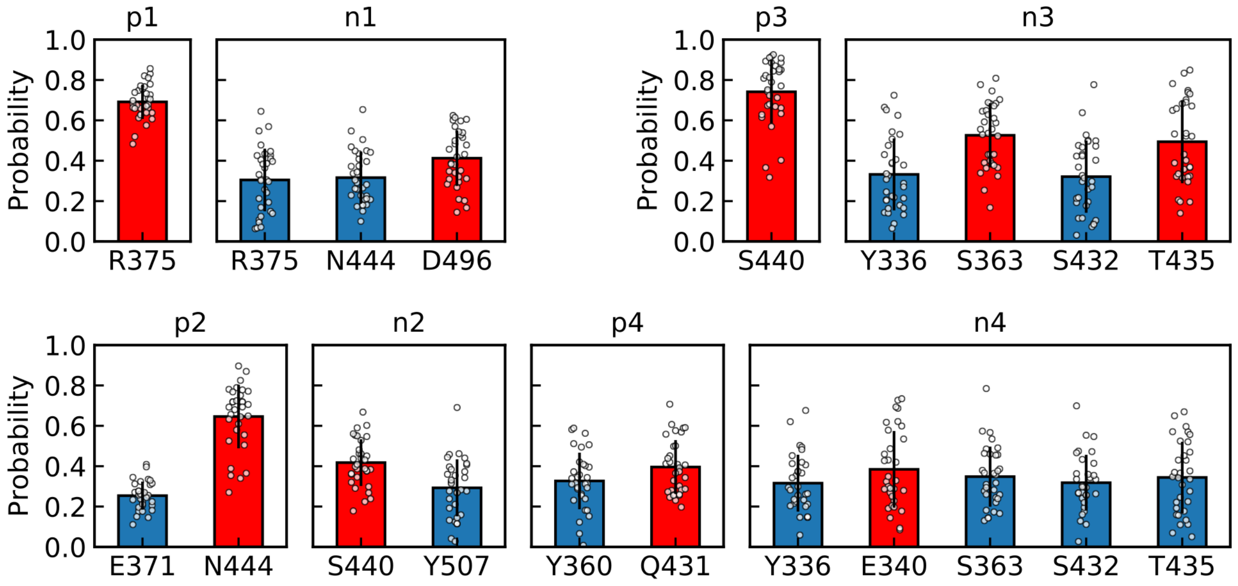

Supplementary Fig. 17: **Protein–lipid interactions define the arrangement of lipid headgroups within the TMEM16K subunit cavity.** Probability that the side chain oxygen or nitrogen atoms of hydrophilic residues are in a direct contact (distance of < 5 Å) with either phosphorus or nitrogen atoms of POPC headgroups at their localization sites (p1, n1, p2, n2, p3, n3, p4, and n4) within the TMEM16K subunit cavity. Only residues with a contact probability of > 20 % are shown. Residues with a contact probability of > 35 % are shown in red and all others are shown in blue. Bars show mean probability, and error bars represent the standard deviation. Data correspond to n=32 independent protomers.

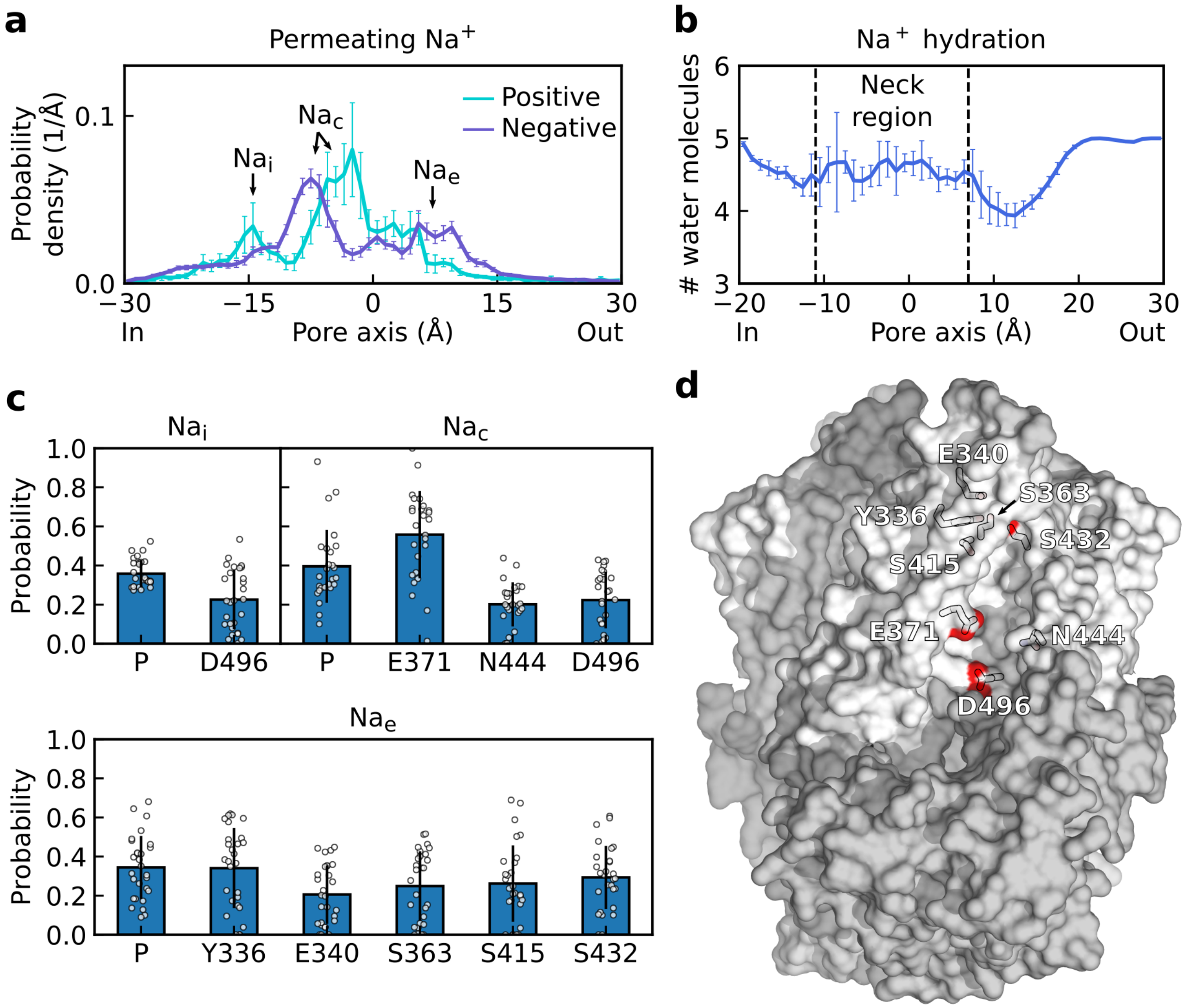

Supplementary Fig. 18: **Analysis of permeating Na^+ ions in the TMEM16K pore. a** Probability density distributions of permeating Na^+ ions along the TMEM16K pore. Data were derived from n=9 and n=14 independent protomers at positive and negative voltages, respectively. **b** Distribution of number of water molecules within the first hydration shell of Na^+ along the TMEM16K pore. Dashed lines indicate the neck region of the pore. Data were derived from n=28 independent protomers. **a**,**b** Distributions were calculated with respect to the pore center. Average distributions are shown and error bars represent the standard error of mean. **c** Probability that an oxygen or nitrogen atom of a hydrophilic residue or phosphorus or nitrogen atom of a POPC headgroup is in direct contact (distance of < 5 Å) with a permeating Na^+ at the Na_i, Na_c, and Na_e sites. Only residues with a contact probability of > 20 % are shown. Bars show mean probability, and error bars represent the standard deviation. Data correspond to n=28, n=26, and n=28 independent protomers for Na_i, Na_c, and Na_e sites, respectively. **d** Positions of residues indicated in **c** are shown within the TMEM16K subunit cavity structure.

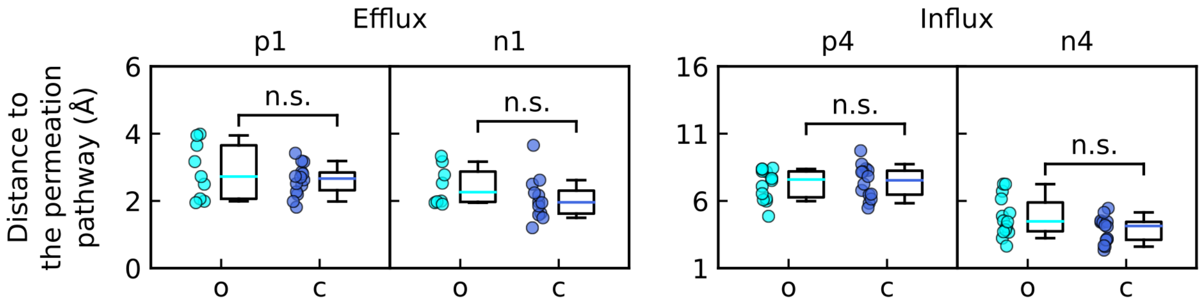

Supplementary Fig. 19: **Lipid headgroups do not dynamically block Na^+ ions permeating through TMEM16K.** Minimum distance between the Na^+ permeation pathway and the nitrogen or phosphorus atoms of POPC headgroups in the plane of the membrane during ion visit to a certain region. Distances were measured separately for the open (o) and closed (c) states of the pore. Each data point represents an independent protomer, and boxplots are defined as follows: the middle line is the median, the lower and upper hinges correspond to the first and third quartiles, whiskers show the 5th and 95th percentiles. Significance was evaluated with the Mann-Whitney test, one-sided: $p > 0.05$ (n.s.). The number of data points and p-values (n_o, n_c, p) for each panel (left to right) were (9, 14, 0.3), (8, 12, 0.08), (14, 14, 0.4), and (14, 14, 0.5).

Supplementary Table 1: **Summary of 4WIS-based nhTMEM16 systems**. Each system with no voltage gave rise to several CompEL replicas. Values for the charge imbalance (dQ) and resulting transmembrane voltage (V) are indicated.

nhTMEM16 (4WIS)

		no voltage		CompEL				
membrane	system	simulation time (ns)	[NaCl] (mM)	replica	[NaCl] (mM)	dQ	V (mV)	simulation time (ns)
PC	s1	2000	250	1	250	14	304 ± 34	1000
				2	250	20	397 ± 29	500
				3	250	28	533 ± 38	1000
				4	250	30	524 ± 36	400
				5	250	22	509 ± 26	500
				6	250	22	478 ± 32	500
	s2	1000	250	1	250	24	471 ± 29	500
				2	250	26	488 ± 37	500
	s3	1000	250	1	250	16	348 ± 28	500
				2	250	24	460 ± 38	500
	s4	1000	250	1	250	20	453 ± 33	500
PC:PS	s1	1000	250	1	250	14	292 ± 28	500
				2	250	24	502 ± 42	500
				3	250	28	659 ± 36	500
				4	1000	24	559 ± 41	500
				5	1000	28	539 ± 51	500
	s2	1000	250	1	250	20	427 ± 32	500
				2	250	22	442 ± 31	500
	s3	1000	250	1	250	20	480 ± 32	500
	s4	1000	1000	1	1000	22	484 ± 44	500
				2	1000	24	550 ± 25	500
PE:PG	s1	1500	250	1	250	22	480 ± 28	500
				2	250	28	568 ± 48	500
	s2	1000	250	1	250	20	422 ± 45	500
				2	250	26	596 ± 37	500
	s3	1000	250	1	250	24	543 ± 30	500
				2	250	26	589 ± 32	500

Supplementary Table 2: **Summary of 6QMA-based (intermediate state) nhTMEM16 systems**. Each system with no voltage gave rise to several CompEL replicas. Values for the charge imbalance (dQ) and resulting transmembrane voltage (V) are indicated.

		no voltage		CompEL				
membrane	system	simulation time (ns)	[NaCl] (mM)	replica	[NaCl] (mM)	dQ	V (mV)	simulation time (ns)
POPC	s1	1000	250	1	250	16	481 ± 30	500
				2	250	18	549 ± 27	500
				3	250	20	518 ± 36	500
				4	250	24	660 ± 35	500
				5	250	28	727 ± 68	500
	s2	1000	250	1	250	16	511 ± 24	500
				2	250	18	515 ± 27	500
				3	250	20	585 ± 34	500
				4	250	22	646 ± 27	500
				5	250	24	701 ± 35	500

Supplementary Table 3: **Summary of the 6OY3-based (L302A mutant of nhTMEM16) systems**. Each system with no voltage gave rise to several CompEL replicas. Values for the charge imbalance (dQ) and resulting transmembrane voltage (V) are indicated.

		no voltage		CompEL				
membrane	system	simulation time (ns)	[NaCl] (mM)	replica	[NaCl] (mM)	dQ	V (mV)	simulation time (ns)
POPC	s1	500	250	1	250	16	472 ± 19	500
				2	250	18	484 ± 25	500
				3	250	20	555 ± 25	500
				4	250	22	627 ± 31	500

Supplementary Table 4: **Summary of TMEM16F systems**. Values for the charge imbalance (dQ) and resulting transmembrane voltage (V) are indicated.

		no voltage		CompEL				
membrane	system	simulation time (ns)	[NaCl] (mM)	replica	[NaCl] (mM)	dQ	V (mV)	simulation time (ns)
POPC	s1	1000	250	1	250	30	976 ± 32	500

Supplementary Table 5: **Summary of the observed scrambling events**. Scrambling events were observed only in the simulations of nhTMEM16 in the fully open state, with the protein embedded in a POPC membrane. The orientation angle was measured for a transported lipid headgroup, when its phosphorus atom occupied the central accumulation site.

voltage polarity	positive			negative		
scrambling direction	down	down	up	up	up	up
orientation angle	28.4	20.7	25.8	140.9	131.4	142.4

Supplementary Table 6: **Selectivity of nhTMEM16 and TMEM16K proteolipidic pores under different conditions.**

protein	nhTMEM16					TMEM16K
membrane	POPC	POPE:POPG	POPC:POPS			POPC
NaCl concentration	250 mM	250 mM	250 mM	1000 mM	200 mM	250 mM
Ca^{2+} concentration	0 mM	0 mM	0 mM	0 mM	∼1 mM	0 mM
P_{Na}/P_{Cl} (V+)	1	9.6	41	31	1.6	$P_{Cl} = 0$
P_{Na}/P_{Cl} (V-)	0.1	6.8	6	0.6	1.3	4.2
P_{Na}/P_{Cl} (both)	1.3	8.7	14	0.7	1.4	4.3

Supplementary Table 7: **Summary of the 4WIS-based nhTMEM16 systems with POPC:POPS (1:1) membrane and 1 mM $CaCl_2$.** Each system with no voltage gave rise to several CompEL replicas. Values for the charge imbalance (dQ) and resulting transmembrane voltage (V) are indicated.

		no voltage		CompEL				
membrane	system	simulation time (ns)	[NaCl] (mM)	replica	[NaCl] (mM)	dQ	V (mV)	simulation time (ns)
PC:PS	s1	100	200	1	200	22	551 ± 45	500
				2	200	22	629 ± 36	500
				3	200	22	605 ± 44	500
				4	200	22	654 ± 34	500
				5	200	22	654 ± 35	500
				6	200	22	651 ± 36	500

Supplementary Table 8: **Summary of TMEM16K systems**. Each system with no voltage gave rise to several CompEL replicas. Values for the charge imbalance (dQ) and resulting transmembrane voltage (V) are indicated.

		no voltage		CompEL				
membrane	system	simulation time (ns)	[NaCl] (mM)	replica	[NaCl] (mM)	dQ	V (mV)	simulation time (ns)
POPC	s1	1000	250	1	250	10	250 ± 29	1000
				2	250	14	362 ± 38	500
				3	250	18	470 ± 34	500
				4	250	24	607 ± 41	500
	s2	1000	250	1	250	16	426 ± 26	500
				2	250	20	490 ± 34	500
				3	250	24	588 ± 44	500
