# Supplementary material for: Molecular mechanisms of ion conduction and ion selectivity in TMEM16 lipid scramblases
Source: Nat Commun. 2021 May 14;12:2826. doi: 10.1038/s41467-021-22724-w (PMC8121942; doi:10.1038/s41467-021-22724-w)
Supplement: Supplementary file 3 — Reporting Summary [file 41467_2021_22724_MOESM3_ESM.pdf]

nature research

Corresponding author(s): Jan-Philipp Machtens

Last updated by author(s): 2020-18-09

Reporting Summary

Nature Research wishes to improve the reproducibility of the work that we publish. This form provides structure for consistency and transparency in reporting. For further information on Nature Research policies, see our Editorial Policies and the Editorial Policy Checklist.

Statistics

For all statistical analyses, confirm that the following items are present in the figure legend, table legend, main text, or Methods section.

n/a	Confirmed	
☐	☒	The exact sample size (n) for each experimental group/condition, given as a discrete number and unit of measurement
☐	☒	A statement on whether measurements were taken from distinct samples or whether the same sample was measured repeatedly
☐	☒	The statistical test(s) used AND whether they are one- or two-sided<br>*Only common tests should be described solely by name; describe more complex techniques in the Methods section.*
☒	☐	A description of all covariates tested
☒	☐	A description of any assumptions or corrections, such as tests of normality and adjustment for multiple comparisons
☐	☒	A full description of the statistical parameters including central tendency (e.g. means) or other basic estimates (e.g. regression coefficient) AND variation (e.g. standard deviation) or associated estimates of uncertainty (e.g. confidence intervals)
☐	☒	For null hypothesis testing, the test statistic (e.g. F, t, r) with confidence intervals, effect sizes, degrees of freedom and P value noted<br>*Give P values as exact values whenever suitable.*
☒	☐	For Bayesian analysis, information on the choice of priors and Markov chain Monte Carlo settings
☒	☐	For hierarchical and complex designs, identification of the appropriate level for tests and full reporting of outcomes
☒	☐	Estimates of effect sizes (e.g. Cohen's d, Pearson's r), indicating how they were calculated

Our web collection on statistics for biologists contains articles on many of the points above.

Software and code

Policy information about availability of computer code

Data collection	Protein models were constructed from the original structures with Modeller version 9.18 (available at https://salilab.org/modeller). Molecular dynamics simulations were conducted with GROMACS version 2016 and 2018 (available at http://manual.gromacs.org).
Data analysis	Visualization was done with PyMol version 1.8 (available at https://pymol.org). Density maps were obtained with GROmaps (available at https://mptg-cbp.github.io/gromaps.html). Other analyses were done with standard GROMACS tools (available at http://manual.gromacs.org) and in-house python scripts (provided as supplementary material) using MDAnalysis library (available at https://www.mdanalysis.org).<br>The python script to analyze ion conduction is freely available at https://jugit.fz-juelich.de/computational-neurophysiology/scramblase_ion_conduction. Sequence alignment was done in Jalview (available at https://www.jalview.org) using ClustalW program (available at http://www.clustal.org/clustal2).

For manuscripts utilizing custom algorithms or software that are central to the research but not yet described in published literature, software must be made available to editors and reviewers. We strongly encourage code deposition in a community repository (e.g. GitHub). See the Nature Research guidelines for submitting code & software for further information.

Data

Policy information about availability of data

All manuscripts must include a data availability statement. This statement should provide the following information, where applicable:

- Accession codes, unique identifiers, or web links for publicly available datasets
- A list of figures that have associated raw data
- A description of any restrictions on data availability

The datasets derived from MD simulations and underlying the figures are available in a Source Data file. Raw data are archived at at Jülich Supercomputer Center and RWTH Aachen University and are available from the corresponding author upon reasonable request.

Field-specific reporting

Please select the one below that is the best fit for your research. If you are not sure, read the appropriate sections before making your selection.

- [x] Life sciences
- [ ] Behavioural & social sciences
- [ ] Ecological, evolutionary & environmental sciences

For a reference copy of the document with all sections, see nature.com/documents/nr-reporting-summary-flat.pdf

Life sciences study design

All studies must disclose on these points even when the disclosure is negative.

Sample size	We increased the sample size (the number of simulation replicas; listed in Supplementary tables 1-6) as much as we can to ensure that the differences between the results of different simulation systems are statistically significant according to the standard error of the mean.
Data exclusions	The simulated protomers with artificially dialated pore (more than 45 water molecules in the extracellular part of the pore) were excluded from the analysis as stated in the Methods section.
Replication	The CompEL systems with nhTMEM16 and TMEM16K simulated in this study were replicated from 4 to 11 times depending on the condition used. More details on the replicas can be found in Supplementary tables 1-6. All replications were successful.
Randomization	No randomization was performed. This is not relevant to our work as we are doing computer simulations.
Blinding	This is not relevant to our work. We are doing computer simulations, and all parameters are set up by the researcher.

Reporting for specific materials, systems and methods

We require information from authors about some types of materials, experimental systems and methods used in many studies. Here, indicate whether each material, system or method listed is relevant to your study. If you are not sure if a list item applies to your research, read the appropriate section before selecting a response.

Materials & experimental systems

n/a	Involved in the study
☒	☐ Antibodies
☒	☐ Eukaryotic cell lines
☒	☐ Palaeontology and archaeology
☒	☐ Animals and other organisms
☒	☐ Human research participants
☒	☐ Clinical data
☒	☐ Dual use research of concern

Methods

n/a	Involved in the study
☒	☐ ChIP-seq
☒	☐ Flow cytometry
☒	☐ MRI-based neuroimaging
